# Supplementary material for: The interactome of intact mitochondria by cross-linking mass spectrometry provides evidence for coexisting respiratory supercomplexes
Source: Mol Cell Proteomics. 2017 Dec 8;17(2):216–32. doi: 10.1074/mcp.RA117.000470 (PMC5795388; doi:10.1074/mcp.RA117.000470)
Supplement: Supplemental Data [file supp_RA117.000470_133922_0_supp_23978_fzffwf.zip › spectra_annotation/mito_spectra_annotation/58-1-4-1-4-1.pdf]

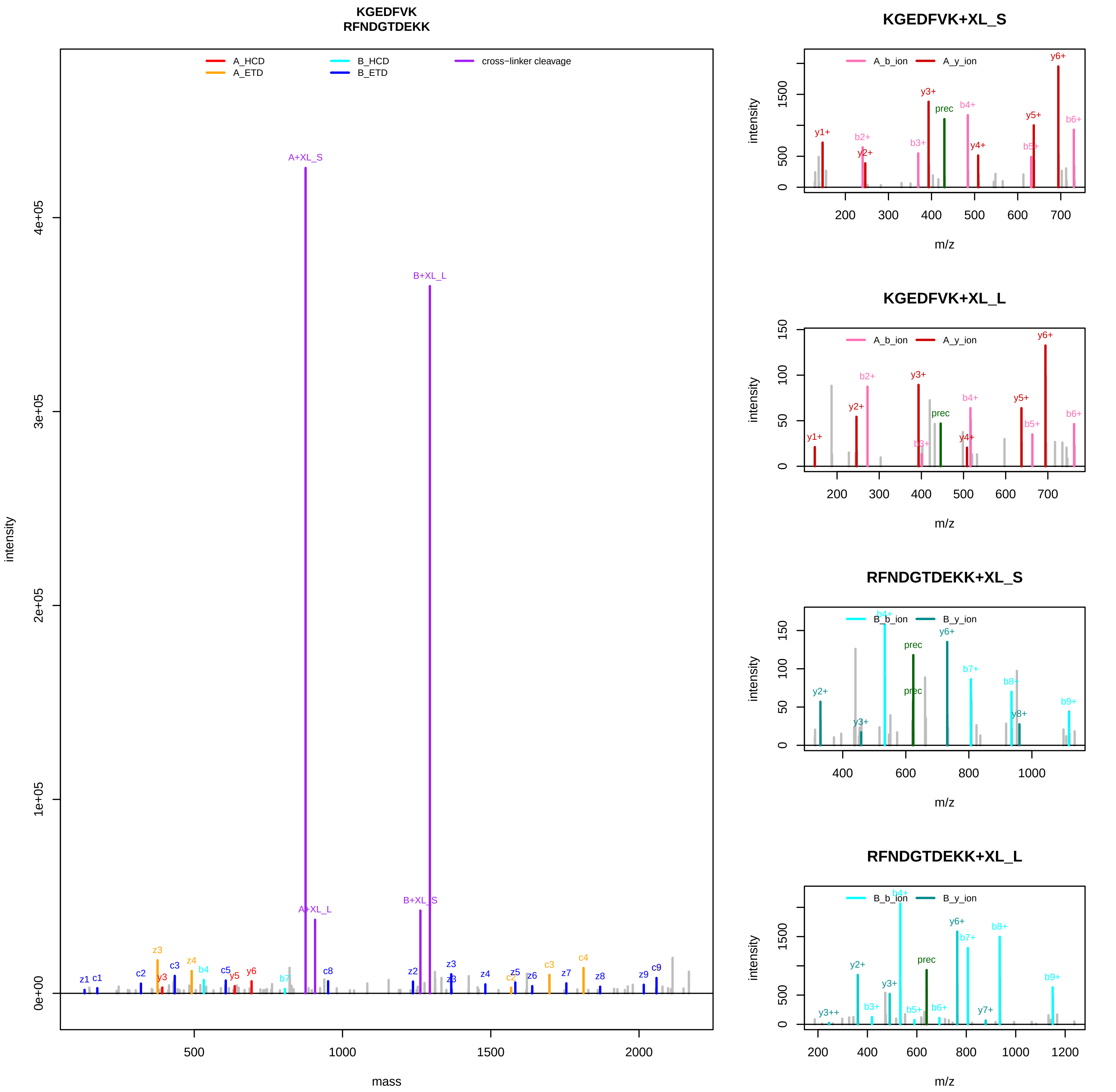

KGEDFVK
RFNDGTDEKK
A_HCD
A_ETD
B_HCD
B_ETD
cross−linker cleavage
A+XL_S
B+XL_L
A+XL_L
B+XL_S
z1 c1 c2 z3 y3 c3 z4 b4 c5 y5 y6 b7 c8 z2 z3 z3 z4 c2 z5 z6 c3 z7 c4 z8 z9 c9
0e+00
1e+05
2e+05
3e+05
4e+05
500
1000
1500
2000
mass
intensity
KGEDFVK+XL_S
A_b_ion
A_y_ion
y1+ b2+ y2+ b3+ y3+ prec b4+ y4+ b5+ y5+ y6+ b6+
m/z
KGEDFVK+XL_L
A_b_ion
A_y_ion
y1+ y2+ b2+ y3+ b3+ prec b4+ y4+ y5+ b5+ y6+ b6+
m/z
RFNDGTDEKK+XL_S
B_b_ion
B_y_ion
y2+ y3+ b4+ prec prec y6+ b7+ b8+ y8+ b9+
m/z
RFNDGTDEKK+XL_L
B_b_ion
B_y_ion
y3++ y2+ b3+ y3+ b4+ b5+ prec b6+ y6+ b7+ y7+ b8+ b9+
m/z
intensity
